# *GhWRKY1-like*, a WRKY transcription factor, mediates drought tolerance in *Arabidopsis* via modulating ABA biosynthesis

**DOI:** 10.1186/s12870-021-03238-5

**Published:** 2021-10-08

**Authors:** Qin Hu, Chuanwei Ao, Xiaorui Wang, Yanfei Wu, Xuezhu Du

**Affiliations:** State Key Laboratory of Biocatalysis and Enzyme Engineering, School of Life Sciences, Hubei University, Wuhan, 430062 Hubei China

**Keywords:** Drought tolerance, ABA, Transcriptional regulation, WRKY, NCED

## Abstract

**Background:**

Drought stress has great negative effects on the plant growth and development. The tolerance of plants to such abiotic stress is triggered by complicated and multilayered signaling pathways to restore cellular homeostasis and to promote survival. The WRKY family is one of the largest transcription factor families in higher plants, and has been well recognized for the roles in regulating plants tolerance to abiotic and biotic stress. However, little is known about how the WRKY genes regulate drought resistance in cotton.

**Results:**

In this work, we identified the WRKY transcription factor *GhWRKY1-like* from upland cotton as a positive regulator of tolerance to drought that directly manipulates abscisic acid (ABA) biosynthesis. Overexpression of *GhWRKY1-like* in *Arabidopsis* constitutively activated ABA biosynthesis genes, signaling genes, responsive genes and drought related maker genes, and led to enhanced tolerance to drought. Further analysis has shown that GhWRKY1-like can interact with “W-box” cis-elements of the promoters of *AtNCED2*, *AtNCED5*, *AtNCED6* and *AtNCED9* which are essential enzymes for ABA biosynthesis, and promotes the expression of those target genes.

**Conclusions:**

In summary, our findings suggest that *GhWRKY1-like* may act as a positive regulator in *Arabidopsis* tolerance to drought via directly interacting with the promoters of *AtNCED2*, *AtNCED5*, *AtNCED6* and *AtNCED9* to promote ABA biosynthesis.

**Supplementary Information:**

The online version contains supplementary material available at 10.1186/s12870-021-03238-5.

## Background

Plants are constantly threatened by various biotic and abiotic stresses in the terrestrial phyllosphere, which cause massive yield and quality losses annually [1, 2]. Drought is a major factor restricting plant growth, survival and yield all over the world [3, 4]. Due to the never stopped co-evolution and mutual selection, plants have evolved a sophisticated surveillance system to perceive water deficit, and to launch prompt defense responses [5–7].

The plant hormone abscisic acid (ABA) is known as an essential phytohormone that positive regulates plant cellular adaptation to drought [8]. When plants are exposed to drought condition, elevation of ABA content is usually seen within a few minutes or hours, leading to stomatal closure and activation of drought responses related genes [9, 10]. ABA is produced from xanthophylls, the cleavage of cis-isomers of xanthophylls by 9-cis-epoxycarotenoid dioxygenases (NCED) into xanthoxin is the first committed step. A short-chain dehydrogenase/reductase encoded by ABA2, catalyzes the oxidation of xanthoxin to abscisic aldehyde which is then converted into ABA by an abscisic aldehyde oxidase encoded by AAO3 [11]. The ABA perception and signaling transduction consist of three families of proteins, including ABA receptors encoded by pyrabactin resistance 1/pyrabactin resistance 1-like/regulatory component of ABA receptor (PYR/PYL/RCAR) genes, negative regulators encoded by type 2C protein phosphatases (PP2Cs) genes, and positive regulators encoded by sucrose non-fermenting 1-related protein kinases [10, 12]. In the absence of ABA, PYR/PYL/RCARs are not bound to PP2Cs, resulting in high PP2C activity, thereby preventing the activation of SnRK2s. ABA perception leads to the conformational change of PYR/PYL/RCAR receptors, which bind to PP2Cs, thereby releasing phosphorylated SnRK2s for subsequent phosphorylation of ABA-responsive element (ABRE) binding factors (ABFs) to regulate transcription of ABA-responsive genes [13–15]. Although, the biosynthesis pathway, perception model and signaling transduction of ABA have been clearly identified, and already uncovered part of the components’ role in plant responses to drought stress, the factors that involved in transducing drought perception to ABA accumulation are unclear.

The WRKY transcription factors is one of the largest transcription factor families in plants [16, 17]. The WRKY family proteins are characterized by one or two highly conserved WRKYGQK heptapeptide at N-terminal and an atypical zinc finger-like motif at its C-terminal [18]. Generally, the WRKY family proteins are divided into 3 groups (I, II, and III) based on the number of the WRKY domains and the type of Zn-finger motif. The Group I WRKY proteins contain two WRKY domains with C2H2-type zinc finger; The Group II and III contain only one WRKY domain, with group II harboring the C2H2 zinc finger, while the group III harboring the C2HC-type zinc finger [18]. Since the first WRKY gene was cloned in sweet tomato, more members of WRKY family were isolated and widely reported to participate in plant response to biotic and abiotic stresses [19–23]. For example, overexpression of *GhWRKY91* (from *Gossypium hirsutum*) enhances drought tolerance in *Arabidopsis* [24]. *AtWRKY46* regulates development, stress and hormonal response by facilitating growth of lateral roots in salt stress through ABA signaling and auxin homeostasis in *Arabidopsis* [25]. Three *Arabidopsis* group III WRKY transcription factors, WRKY46, WRKY54, and WRKY70, are involved in both BR-regulated plant growth and drought response, the wrky46 wrky54 wrky70 triple mutant shows defects in BR-regulated growth,but is more tolerant to drought stress [26]. Overexpression of *GhWRKY27a* reduces tolerance to drought and resistance to *Rhizoctonia solani* infection in transgenic tobacco [27].

Cotton is one of the most important economic crops and is cultivated globally [10]. Cotton production is limited by various abiotic stresses, especially drought stress, which causes substantial loss of cotton yield [10]. Therefore, it is meaningful to elucidate the molecular mechanism how cotton copes with drought stress. Here, we identified a Group I WRKY transcription factor *GhWRKY1-like*, which was obviously up-regulated by mannitol, dehydration and NaCl treatment, overexpression WRKY enhances tolerance to drought stress with hyperaccumulated ABA content and activated ABA-dependent stress responses in *Arabidopsis*. Further experiments demonstrated that the key enzymes involved in ABA biosynthesis *NCED3*, *NCED5*, *NCED6* and *NCED9* were the direct target of *GhWRKY1-like*, and were significantly up regulated by GhWRKY1-like. Our results suggest that *GhWRKY1-like* may promotes plant tolerance to drought stress via directly manipulating ABA de novo biosynthesis.

## Results

### GhWRKY1-like is nuclear-localized protein and function as a transcription factor

**Fig. 1:**
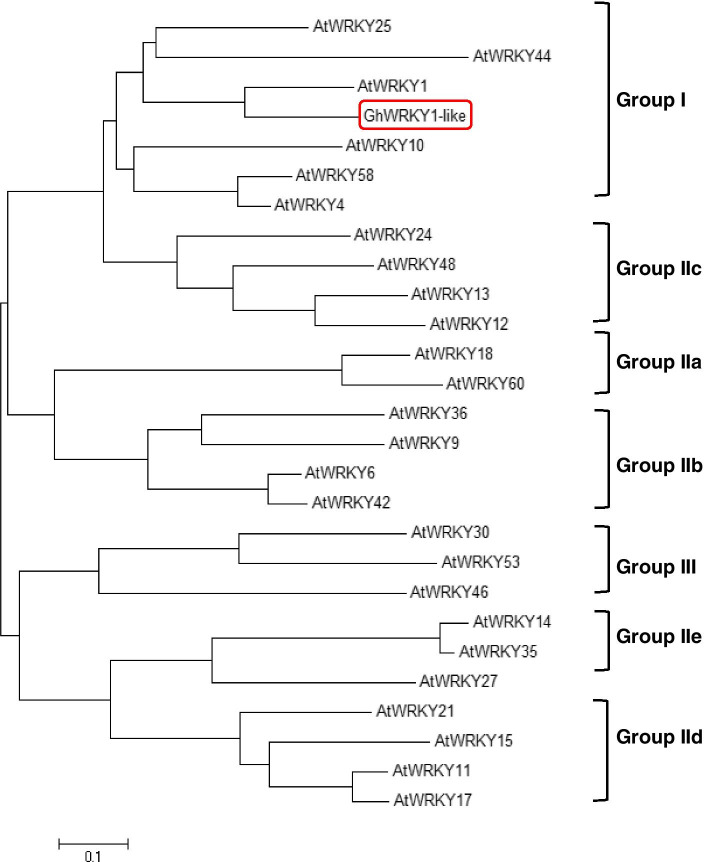
Phylogenetic relationship of WRKY proteins from *Arabidopsis* and *Gossypium hirsutum*. All the 27 WRKYs protein sequences were subjected to Clustal W using the neighbor-joining method in MEGA 6 and can be divided into three groups (I, II, III), and Group II is further divided into five subgroups (IIa, IIb, IIc, IId, IIe). All the WRKY proteins used for the phylogenetic tree are: AtWRKY1 (AEC05881), AtWRKY4 (NP_172849), AtWRKY6 (NP_564792), AtWRKY9 (AEE34757), AtWRKY10 (NP_175956), AtWRKY11 (NP_849559), AtWRKY12 (NP_566025), AtWRKY13 (NP_195651), AtWRKY14 (NP_564359), AtWRKY15 (NP_973521), AtWRKY17 (NP_565574), AtWRKY18 (NP_567882), AtWRKY21 (NP_565703), AtWRKY24 (NP_198972), AtWRKY25 (NP_180584), AtWRKY27 (NP_568777), AtWRKY30 (NP_568439), AtWRKY35 (AEC09027), AtWRKY36 (NP_564976), AtWRKY42 (NP_192354), AtWRKY44 (NP_181263), AtWRKY46 (AEC10690), AtWRKY48 (NP_199763), AtWRKY53 (NP_194112), AtWRKY58 (NP_186757), AtWRKY60 (NP_180072), and GhWRKY1-like (XP_016696352). At, *Arabidopsis thaliana*; Gh, *Gossypium hirsutum*

**Fig. 2:**
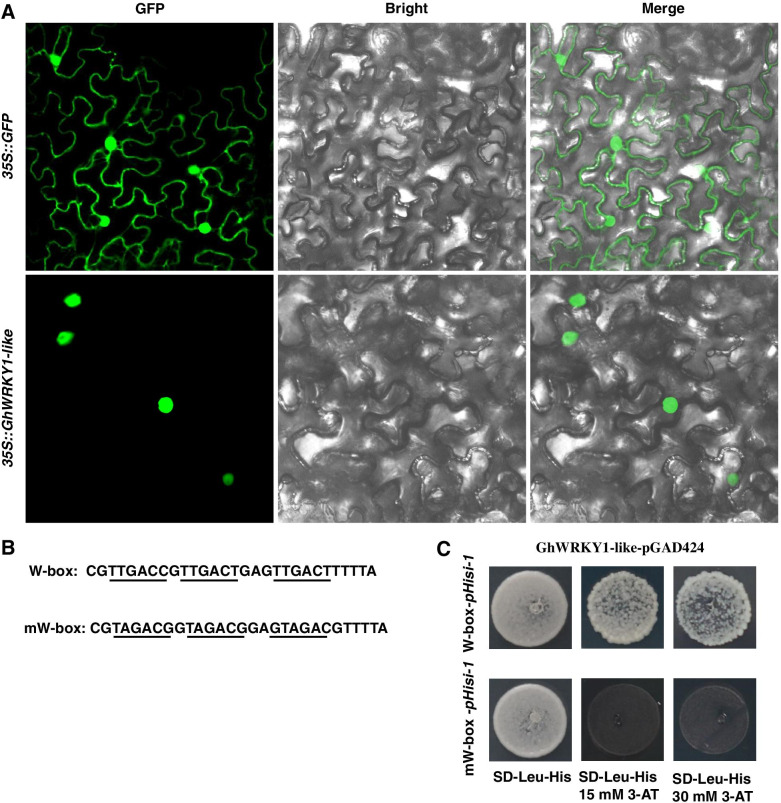
Characterization of GhWRKY1-like as a WRKY family transcriptional factor. **A** Subcellular localization of GhWRKY1-like protein. GFP fluorescence of *35S::GFP* and *35S::GFP-GhWRKY1-like* were observed in the tobacco epidermal cell using confocal microscope. **B** The sequence of the triple tandem repeats of the W-box and mutant W-box binding elements. **C** Yeast one-hybrid assay using the triple tandem repeats of the W-box and mW-box as bait. Yeast cells were grown on SD-Leu-His containing different concentrations of 3-amino-1,2,4-triazole (3-AT) for 3 to 5 d at 30 °C

Previously, we have identified a WRKY transcription factor *GhWRKY1-like* as a positive regulator in cotton defense against *Verticillium dahliae* [28]. The *GhWRKY1-like* gene contains a complete open reading frame (ORF) of 1215 bp that encodes a protein with 404 amino acids. Alignment analysis of GhWRKY1-like protein sequence with its homologous sequences, including AtWRKY1, GmWRKY1, PtWRKY1 and TcWRKY1 was performed and the result showed that *GhWRKY1-like* contains two WRKY domain (WRKYGQK) and two C_2_H_2_ zinc finger (C-X_4–5_-CX_22–23_-H-X_1_-H) motif (Fig. S1). Phylogenetic analysis performed with the AtWRKY1 protein sequences showed that the closest orthologs of GhWRKY1-like is AtWRKY1, and belongs to group I WRKY transcription factor (Fig. 1). To analysis the subcellular localization of GhWRKY1-like, the protein of GhWRKY1-like was fused with C-terminal GFP protein, and transient expressed in *Nicotiana benthamiana* leaves. The result showed that the GhWRKY1-like-GFP fluorescence was seen mainly in the nuclei of cells, indicating GhWRKY1-like is a nuclear-localized protein (Fig. 2 A).

Existing research suggests that WRKY transcription factor reprogram gene(s) expression level by directly binding to the W-box [(C/T)TGAC(C/T)] or W-box like [TGAC(C/T)] cis-regulatory elements in the promoter regions of downstream target genes [29]. Thus, we detected whether GhWRKY1-like protein has the ability to bind to the W-box sequence by yeast one-hybrid assay. The triple tandem repeat sequences of W-box (TTGACT) and triple tandem repeat sequences of mutant W-box (TAGACG) were inserted into the pHisi-1 vector to generate W-box-pHisi-1 and mW-box-pHisi-1 constructs, respectively (Fig. 2 B). The full length of GhWRKY1-like was amplified and inserted into pGAD424 vector to form a yeast effector vector GhWRKY1-like-pGAD424. The results confirmed the interaction between GhWRKY1-like-pGAD424 and W-box-pHisi-1, but not occurred between GhWRKY1-like-pGAD424 and mW-box-pHisi-1 (Fig. 2 C). Above findings implied that GhWRKY1-like was a WRKY transcription factor and could bind to the W-box elements of the target promoters to modulate expression of downstream genes.

### Overexpression of GhWRKY1-like enhances transgenic plant sensitivity to ABA and tolerance to mannitol

**Fig. 3:**
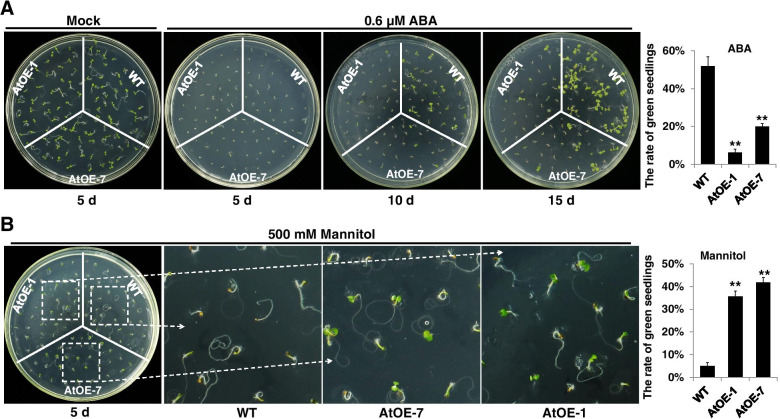
Overexpression of *GhWRKY1-like* causes hypersensitivity to ABA-elicited seed germination and enhanced tolerance to mannitol treatment. **A** Green seedling rate of WT and *GhWRKY1-like* transgenic lines on 1/2 MS medium supplied with 0.6 μM ABA. Values are means ± SD; *n* = 6. Comparisons were performed with Student’s *t* test. **, *P* < 0.01. **B** Green seedling rate of WT and *GhWRKY1-like* transgenic lines on 1/2 MS medium supplied with 500 mM mannitol. Values are means ± SD; n = 6. Comparisons were performed with Student’s *t* test. **, *P* < 0.01

Previous study has shown that *GhWRKY1-like* is ubiquitously expressed in several organs, and is significantly up-regulated by *V. dahliae* treatment. Furthermore, we found that *GhWRKY1-like* was obviously up-regulated by mannitol, dehydration and NaCl treatment in both roots and leaves in upland cotton cv YZ1 using reverse transcription quantitative PCR (RT-qPCR), but GhWRKY1-like showed no response to ABA treatment (Fig. S2A). The induce expression patter suggested that *GhWRKY1-like* might also involved in plant response to abiotic stress (Fig. S2B). To elucidate the putative biofunction of *GhWRKY1-like* in plant abiotic stress tolerance, the *GhWRKY1-like* was introduced into *Arabidopsis thaliana* for ectopic expression. According to the expression level, we choose one high expression line (*OE-1*) and one moderate expression line (*OE-7*) for further research (Fig. S2C). The T4 generation homozygous transgenic seeds of *OE-1* and *OE-7* was subjected to ABA and mannitol treatment to observe the sensitivity to ABA and mannitol by calculating the germination rate or green seedling rate (Fig. 3). The results showed that the germination rate or green seedling rate of *GhWRKY1-like* over-expression transgenic *Arabidopsis* lines were similar to that of WT under normal conditions, while compared with WT, the seed germination rate of the *GhWRKY1-like* over-expression transgenic *Arabidopsis* lines were significantly decreased with ABA treatment (Fig. 3 A), and the green seedling rate of *GhWRKY1-like* over-expression transgenic *Arabidopsis* lines were significantly increased with mannitol treatment (Fig. 3 B). These findings indicated that *GhWRKY1-like* may promotes plant drought tolerance via ABA signaling pathway.

### Overexpression of GhWRKY1-like enhances transgenic plant tolerance to drought

**Fig. 4:**
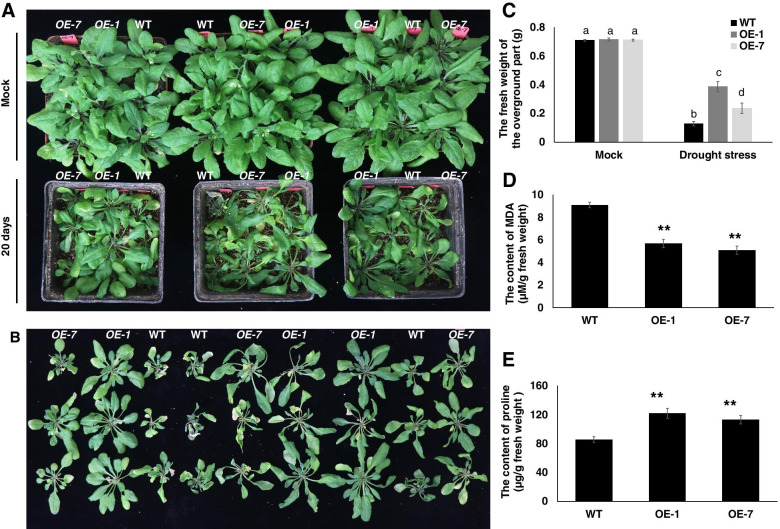
Overexpression of *GhWRKY1-like* conferred drought tolerance in *Arabidopsis*. **A** The phenotypes of *GhWRKY1-like* transgenic lines tolerant to drought stress. **B** The size of WT and *GhWRKY1-like* transgenic lines after 20 days without watering. **C** The fresh weight of WT and *GhWRKY1-like* transgenic lines after 20 days without watering. Values are means ± SD; *n* = 18. In each column, values not followed by the same letters are significantly different based on Tukey’s multiple comparison test (*P* < 0.05). **D** The MAD content of WT and *GhWRKY1-like* transgenic lines after 20 days without watering. Values are means ± SD; n = 6. Comparisons were performed with Student’s *t* test. **, *P* < 0.01. **E** The proline content of WT and *GhWRKY1-like* transgenic lines after 20 days without watering. Values are means ± SD; n = 6. Comparisons were performed with Student’s *t* test. **, *P* < 0.01

To further investigate how *GhWRKY1-like* functions in drought response during vegetative growth of the transgenic plants. Two-week-old vermiculite-grown (watered with Hogland medium) *GhWRKY1-like* transgenic *Arabidopsis* seedlings and WT were kept away from water for 20 days. As showed in Fig. 4 A, the *GhWRKY1-like* over-expression plants were much bigger than that of WT, and the performance of *OE-1* line with higher *GhWRKY1-like* expression level was better than that of *OE-7*, which suggested that drought tolerance mediated by *GhWRKY1-like* was dosage-dependent (Fig. 4 A and B). The results of fresh weight of the transgenic lines and WT under normal conditions or water-withholding conditions also supported this conclusion (Fig. 4 C). Malondialdehyde (MDA) is one of the most important products of cell-membrane lipid peroxidation, and its production can aggravate the membrane damage. Therefore, MDA content is a common indicator to understand the degree of membrane lipid peroxidation and the potential capability of stress tolerance in plants [30]. The MDA content was obviously lower in *GhWRKY1-like* transgenic lines than that of WT (Fig. 4 D), which indicating *GhWRKY1-like* promotes *Arabidopsis* tolerance to drought by suppression MDA accumulation. Proline is a low molecular weight cyclic amino acid and is known to provide osmotic adjustments in plants under stressful environments. Proline equilibrates the intracellular redox homeostasis by stabilizing antioxidant system through osmotic adjustments and protecting the integrity of cell membranes [31]. Meanwhile, we detected the proline content in *GhWRKY1-like* transgenic lines and WT, and the result showed that proline was significantly hyperaccumulated in *GhWRKY1-like* over-expression lines than that of WT (Fig. 4 E), which indicated that *GhWRKY1-like* over-expression lines had stronger resistance capacity to drought stress.

### GhWRKY1-like positively regulates ABA content and ABA-responsive genes in transgenic *Arabidopsis*

**Fig. 5:**
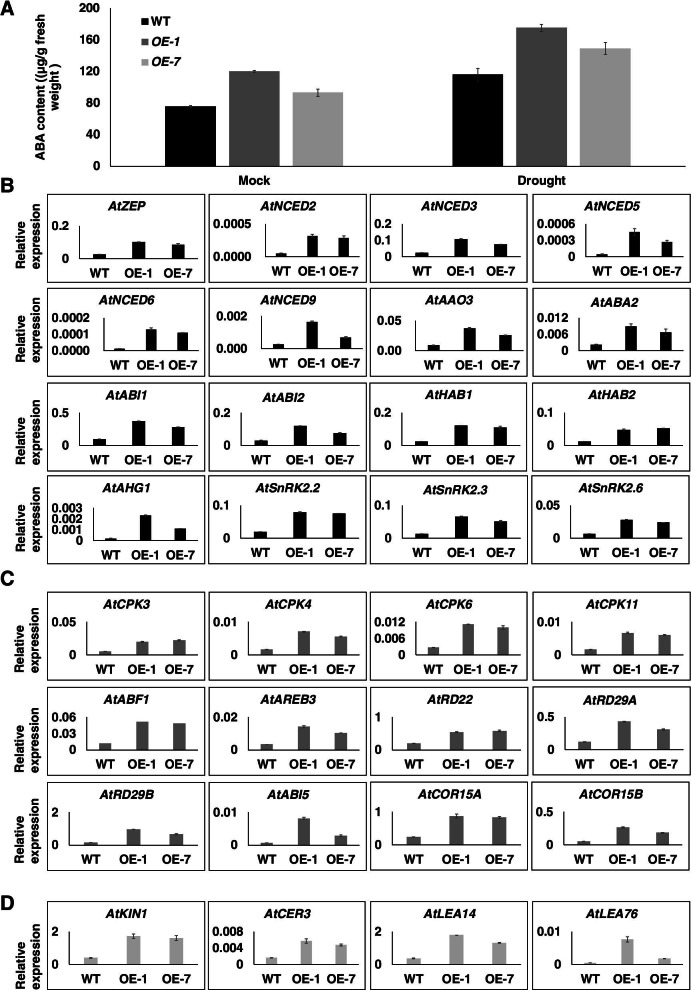
*GhWRKY1-like* positively regulates *Arabidopsis* tolerance to drought via manipulating ABA biosynthesis and ABA mediated drought responses. **A** The ABA content in WT and *GhWRKY1-like* transgenic lines in normal conditions and drought conditions. Values are means ± SD; *n* = 9. Comparisons were performed with Student’s *t* test. *, *P* < 0.05; **, *P* < 0.01. **B** Expression of genes involved in ABA biosynthesis and the ABA signaling pathway in WT and *GhWRKY1-like* transgenic lines after 10 days without watering. The values are normalized to *AtACTIN2* and expressed as the means ± SD; *n* = 3. **C** Expression of genes involved in ABA responsive genes in WT and *GhWRKY1-like* transgenic lines after 10 days without watering. The values are normalized to *AtACTIN2* and expressed as the means ± SD; n = 3. **D** Expression of genes involved in abiotic stress-responsive genes in WT and *GhWRKY1-like* transgenic lines after 10 days without watering. The values are normalized to *AtACTIN2* and expressed as the means ± SD; n = 3

The phytohormone abscisic acid (ABA) plays an important role in plant development and adaption to biotic and abiotic stresses. Plants rapidly initiate ABA biosynthesis in various organs in response to water deficit. The activated ABA signaling pathway eventually leads to stomatal closure, osmoprotectants accumulation, phenotypical and physiological adaptations to enhance drought resistance [8]. The induced expression pattern showed that *GhWRKY1-like* had no response to ABA treatment (Fig. S2A and B), but the *GhWRKY1-like* over-expression lines were much sensitively to ABA treatment as indicated by lower seed germination rate (Fig. 3A). These findings implied that GhWRKY1-like may participate in ABA biosynthesis regulation rather than ABA signaling transduction. Thus, we detected the ABA content in *GhWRKY1-like* over-expression lines and WT. In accordance with expectation, the ABA content was obviously increased in *GhWRKY1-like* over-expression lines than that of WT in both normal conditions or drought conditions (Fig. 5A). The expression levels of genes involved in ABA biosynthesis also consistent with this conclusion (Fig. 5B). We also detected the ABA-responsive genes and drought-responsive genes in *GhWRKY1-like* over-expression lines and WT by RT-qPCR (Fig. 5C and D). The expression levels of these genes were consistently significantly up-regulated in the *GhWRKY1-like* over-expression lines (Fig. 5C and D). Above findings suggested that *GhWRKY1-like* was a positive regulator in *Arabidopsis* response to drought via manipulating ABA biosynthesis.

### GhWRKY1-like promotes ABA biosynthesis by directly binding to the promoters of *AtNCED2*, *AtNCED5*, *AtNCED6* and *AtNCED9*

**Fig. 6:**
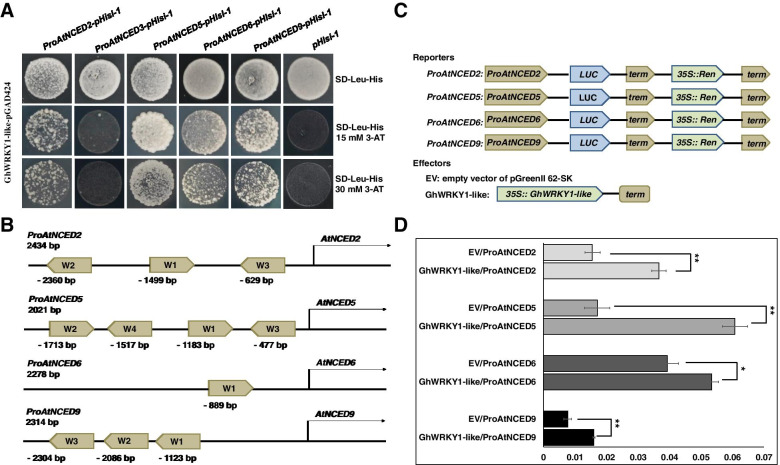
GhWRKY1-like positively regulated the expression of ABA biosynthesis related genes *AtNCED2*, *AtNCED5*, *AtNCED6* and *AtNCED9*. **A** Yeast one-hybrid assay showing the binding of GhWRKY1-like to the promoters of *AtNCED2*, *AtNCED5*, *AtNCED6* and *AtNCED9*. Empty pHisi-1 vector was used as a negative control. Yeast cells were grown on SD-Leu-His containing different concentration different concentrations of 3-amino-1,2,4-triazole (3-AT) for 3 to 5 d at 30 °C. **B** Diagram of W-box positions in the promoters of *AtNCED2*, *AtNCED5*, *AtNCED6* and *AtNCED9*. **C** Diagram of the effector and reporter constructs for the transactivation assay. The reporter carries a renilla luciferase gene under the control of the *35S* promoter and a firefly luciferase reporter gene under the control of the promoter of *AtNCED2*, *AtNCED5*, *AtNCED6* or *AtNCED9*. 35S, promoter of cauliflower mosaic virus 35S RNA gene; REN, Renilla luciferase; CaMV term, cauliflower mosaic virus terminator; LUC, firefly luciferase. **D** Translational changes of the reporters in the presence of GhWRKY1-like in *Arabidopsis* protoplasts. The LUC/REN activity ratios are the means ± SD; n = 3. Comparisons were performed with Student’s *t* test. *, *P* < 0.05; **, *P* < 0.01

To elucidated the mechanism(s) by which *GhWRKY1-like* regulates ABA biosynthesis, we cloned several promoters (about 2000 bp in the upstream of ATG) of ABA biosynthesis related genes to detect the interaction with GhWRKY1-like by yeast one hybrid. As shown in Fig. 6A, we identified that GhWRKY1-like could directly bind to the promoters of *AtNCED2* (nine-cis-epoxycarotenoid dioxygenases, *NCED*; *ProAtNCED2*, 2434 bp in the upstream of ATG), *AtNCED5* (*ProAtNCED5*, 2021 bp in the upstream of ATG), *AtNCED6* (*ProAtNCED6*, 2278 bp in the upstream of ATG) and *AtNCED9* (*ProAtNCED9*, 2314 bp in the upstream of ATG), but not interact with promoter of *AtNCED3* (*ProAtNCED3*, 2200 bp in the upstream of ATG). Sequence analysis showed that there were at least one W-box in the promoter region of *AtNCED2*, *AtNCED5*, *AtNCED6* and *AtNCED9* (Fig. 6B). To elucidate the function of GhWRKY1-like (activator or repressor) for *proAtNCED2*, *proAtNCED5*, *proAtNCED6* and *proAtNCED9*, a dual-luciferase reporter system was performed in *Arabidopsis* protoplasts (Fig. 6C). The ratio of LUC activity to the control REN (Renilla luciferase) activity or the LUC luminescence intensity was used to indicate the activity of promoters. As shown in Fig. 6D, compared with the control vector, the expression levels of *proAtNCED2::LUC*, *proAtNCED2::LUC5*, *proAtNCED6::LUC* and *proAtNCED9::LUC* were significantly activated in the present of GhWRKY1-like (Fig. 6D). These data suggested that *AtNCED2*, *AtNCED5*, *AtNCED6* and *AtNCED9* were the direct targets of GhWRKY1-like, and the accumulated ABA content in *GhWRKY1-like* over-expression lines might resulted from the activation of *AtNCED2*, *AtNCED5*, *AtNCED6* and *AtNCED9* expression.

## Discussion

Global climate models predict a significant increase in intensity and frequency of hot and dry days, which is predicted to have a general negative effect on crop yield and quality [32, 33]. With increasing water scarcity and global population explosion, drought is emerging as a prominent constraint on crop production [10, 34]. Owing to the characteristics of the sessile lifestyle, plants have evolved a complex regulatory circuitry in response to drought stress. The plant hormone ABA regulates various physiological processes throughout plant life cycles and manipulated plant resistant responses to various abiotic stresses. The quickly accumulated ABA content is a typical plants’ response to water deficit and leads to stomatal closure and the expression of numerous stress-responsive genes [35, 36]. The mutant that impaired ABA synthesis or ABA signaling pathway always resulted in hypersensitivity to drought. For example, the AtNCED3 is known as an important enzyme for ABA accumulation during water deficit and the expression level is highly induced in the vascular tissues by drought stress, the knock out mutant of *AtNCED3* (*nced3–2*) results in decreased ABA accumulation and is more sensitive to drought stress [37]. The *aba2* mutant in *Arabidopsis* shows ABA-insensitive phenotype and reduced seed dormancy with high salt concentration, and reduced stomatal closure in response to a decrease in humidity [38]. The *snrk2.2/3/6* triple mutant is totally abolished in ABA response and hypersensitive to water deficit [39, 40]. Although ABA biosynthesis and signaling pathway have been studied extensively, and nearly all enzymes involved in ABA synthesis have been cloned, the mechanism by which plants precisely regulate ABA accumulation (biosynthesis and degradation) after the perception of water deficit or osmotic stress is not well understood.

Actually, the basis for plants to establish effective defense responses depends on the precise expression reprogramming of stress-responsive genes including stress-induced hormones biosynthesis, signaling transduction and osmoprotectants metabolism related genes, and this regulatory circuitry is comprised of transcriptional activators and repressors [41, 42]. The WRKY faminly is one of the largest families of transcription regulators in plants, and have been well recognized for the roles in regulating abiotic and biotic stress tolerance. For instance, ectopic expression of *GhWRKY33* enhances transgenic *Arabidopsis* drought sensitivity with down-regulated expression level of *RD29A*, *DREB2A*, *ERD15*, *SOS2*, *ABI1* and *RAB18* [43]. Interfamily expression of *PbrWRKY53* from *Pyrus betulaefolia* in tobacco and *Pyrus ussuriensis* confers enhanced tolerance to drought stress in the transgenic plants, and exhibits better water status, less reactive oxygen species generation and higher levels of antioxidant enzyme activities and metabolites than the wild type [44]. Further research demonstrates that *PbrWRKY53* can bind to the W-box element in the promoter region of *PbrNCED1* to modulate stomatal aperture and ABA biosynthesis [44]. Conversely, the *Arabidopsis* wrky46wrky54wrky70 triple mutant shows enhanced tolerant to drought stress via modulating brassinosteroids-regulated plant growth and promoting expression levels of drought responsive genes, which indicates AtWRKY46, AtWRKY54 and AtWRKY70 are negative regulators of drought tolerance [26]. In our study, we found that *GhWRKY1-like* was significantly induced by mannitol, dehydration and Nacl treatment, but showed no response to ABA treatment (Fig. S2). Further research confirmed that over-expression *GhWRKY1-like* enhanced *Arabidopsis* transgenic lines resistance to drought and hyposensitive to ABA treatment accompanied with hyperaccumulated ABA content (Figs. 4 and 5). The yeast one hybrid assay and transient expression analysis demonstrated that *GhWRKY1-like* could bind to the W-box element in the promoter region of *AtNCED2*, *AtNCED5*, *AtNCED6* and *AtNCED9*, and could promote the expression level of those genes involved in ABA biosynthesis (Fig. 6). According to present research in Arabidopsis, there are five genes encode the 9-cis-epoxycarotenoid dioxygenases and constitute a key step in the regulation of ABA biosynthesis [45]. AtNCED3 has been shown to play a major role in the regulation of ABA synthesis in response to water deficit [45], whereas *AtNCED6* and *AtNCED9* have been shown to be essential for the ABA production in the embryo and endosperm that imposes dormancy [46]; *AtNCED2*, *AtNCED5*, *AtNCED9* have been shown to contribute to the thermoinhibition of germination by increasing ABA levels at high temperature [47]. A detailed phenotypic analysis of *Atnced* single, double and triple mutants generated from the combination of the *Atnced5* mutation with *At*nced3, *At*nced6 and *At*nced9 showed that *AtNCED5* participates in the regulation of seed dormancy together with *AtNCED6* and *AtNCED9*, and contributes with *AtNCED3* to the increased ABA levels induced in vegetative tissues upon the onset of water deficit [11]. Although, *GhWRKY1-like* not directly binds to the promoter region of *AtNCED3* which play a major role in the regulation of ABA synthesis in response to water deficit (Fig. 6A), *GhWRKY1-like* may indirectly manipulate *AtNCED3* mediated drought responses via directly promoting the transcriptional levels of *AtNCED5*, *AtNCED6* and *AtNCED9* (Fig. 6A). Moreover, the expressions of the ABA signaling genes (*ABI1*, *ABI2*, *ABI4*, and *ABI5*), responsive genes (*RD29A*, *COR15A*, *COR15B*, and *RD22*) and stress-related marker genes (*KIN1*, *LEA14*, *LEA76*, and *CER3*) were significantly up-regulated in transgenic lines under drought stress (Fig. 5B).

## Conclusion

Taken together, the results obtained in this study indicated that *GhWRKY1-like* played a positive role in drought tolerance by promoting ABA biosynthesis via directly regulating *AtNCED2*, *AtNCED5*, *AtNCED6* and *AtNCED9* expression, and thus holding a great potential in improving plant stress tolerance.

## Methods

### Plant material, growth conditions and treatments

Cotton (*Gossypium hirsutum*) cultivar “YZ1” provided by our lab was used to investigated the abiotic stresses induced expression pattern of *GhWRKY1-like* including 300 mM mannitol, 100 μM ABA, 200 mM NaCl and dehydration. Cotton seeds of YZ1 were germinated and cultivated with Hoagland solution [48] in a culture room until the three-leaf stage was reached [28]. Then, the seedings were moved to Hoagland solution containing 300 mM mannitol or 200 mM NaCl for mannitol and NaCl treatment. For ABA treatment, the seedings were moved to Hoagland solution containing 100 μM ABA and the leaves were also sprayed with this solution. For dehydration treatment, the whole plants were removed from the Hoagland solution, and placed on paper towels on a laboratory bench. For each treatment, the roots and leaves were harvested at 0, 3, 6, 12, 24, and 48 h, respectively. Samples were frozen in liquid nitrogen and stored at − 80 °C for further research.

*Arabidopsis thaliana* seeds (Col-0, obtained from the Arabidopsis Biological Resource Center (ABRC) through TAIR (www.arabidopsis.org) and kept in our lab) were firstly vernalized at 4 °C for 2 days, then sowing in soil and the seedlings were grown in a culture room at with a22 °C and 16 h light/8 h dark photoperiod [45]. For ABA and mannitol treatments, the *Arabidopsis* seeds were grown on the 1/2 Murashige and Skoog (MS) agar plate supplied with 0.6 μM ABA or 500 mM mannitol for 2 days at 4 °C, and them move to normal growth condition to observe the green seeding rate.

### RAN extraction and expression analysis

Total RNA extraction, reversing transcription for the first strand cDNA synthesis and quantitative real-time PCR (qRT-PCR) was carried out according to our previous description [28]. The relative gene expression level was calculated by the 2^–ΔΔCT^ method. *GhUB7* and *AtACTIN2* were used as an internal controls for cotton and *Arabidopsis*, respectively. The primer information was listed in Table S1.

### *GhWRKY1-like* cloning, sequence analysis, vector construction and *Arabidopsis* transformation

The isolation and cloning of *GhWRKY1-like* open reading frame (ORF, Gh_D11G1536, https://cottonfgd.org) and the construction of *GhWRKY1-like* over-expression vector were performed as previously described [28], and the *Agrobacterium*-mediated transformation by floral dipping method was performed in *Col-0* Arabidopsis to generate *GhWRKY1-like* over-expression lines [49]. The analysis of amino acid sequences alignment and phylogenetic relationship of the WRKY proteins were performed using ClustalX (http://www.clustal.org) and MEGA6 (http://www.megasoftware.net), respectively.

### Subcellular localization of GhWRKY1-like protein

The ORF of *GhWRKY1-like* were amplified with GhWRKY1-like-GFP-F/R, and cloned into vector pMDC83 by BP and LR recombination reactions to generate the C-terminally fused GFP construct to determine the subcellular localization of GhWRKY1-like protein. The empty vector of pGWB452 with a N-terminally fused GFP was used as control. All vectors were transformed into *Nicotiana benthamiana* leaves via the *Agrobacterium tumefaciens* strain GV3101 [50] and the fluorescence of GFP were observed using a confocal microscope (Olympus FV1200) after infiltrated for 60 h. The primers used in this study are listed in Table S1.

### Yeast one-hybird assay

The yeast one-hybird assay was conducted according to the manufacturer’s protocol (MATCHMAKER One-Hybrid System User Manual, Clontech). The ORF of *GhWRKY1-like* were amplified with *GhWRKY1-like*-AD-F/R and cloned into vector pGAD424 at *EcoRI and BamH* to generate GhWRKY1-like-pGAD424 construct as AD vector. Oligonucleotide sequences containing specific adaptor and the W-box or mW-box were synthesized and inserted into pHisi-1 vector at *SacI* to obtain *W-box-pHisi-1* and *mW-box-pHisi-1* as reporter vectors. The reporter vector was linearized with *XhoI* and co-transferred with GhWRKY1-like-pGAD424 into YM4271 yeast strain. The promoter region of *AtNCED2* (AT4G18350, https://www.arabidopsis.org/), *AtNCED3* (AT3G14440, https://www.arabidopsis.org/), *AtNCED5* (AT1G30100, https://www.arabidopsis.org/), *AtNCED6* (AT3G24220, https://www.arabidopsis.org/) and *AtNCED9* (AT1G78390, https://www.arabidopsis.org/) were also amplified with the corresponding primers and inserted into pHisi-1 vector at *SacI* to obtain *ProAtNCED2-pHisi-1*, *ProAtNCED3-pHisi-1*, *ProAtNCED5-pHisi-1*, *ProAtNCED6-pHisi-1* and *ProAtNCED9-pHisi-1* as reporter vectors. The reporter vector was linearized with *XhoI* (*ProAtNCED3-pHisi-1* and *ProAtNCED9-pHisi-1* were linearized with *AflII*) and co-transferred with GhWRKY1-like-pGAD424 into YM4271 yeast strain. The yeast cells were cultured on synthetic SD-His-Leu medium with 15 mM or 30 mM 3-amino-1,2,4-triazole (3-AT) for 3 to 5 d at 30 °C to confirm the interaction between GhWRKY1-like and each promoter. The primers used in this study are listed in Table S1.

### Drought treatment in transgenic *Arabidopsis*

For drought treatment, the seeds of WT and the T4 generation of *GhWRKY1-like* overexpression lines were sown in vermiculite for 3 weeks and watered with Hoagland solution. The drought stress were set as 20 days without watering. And the overground parts were collected for fresh weight, malondialdehyde (MAD) and proline content determination.

### The determination of MAD and proline content

The measurement of MAD was performed using the thiobarbituric acid (TBA) method [51]. In brief, approximately 100 mg fresh samples were immediately homogenized in 1 mL 5% (w/v) trichloroacetic acid (TCA) solution using a Tissuelyser (Jingxin, Shanghai, China). After centrifugated at 4 °C for 10 min at 6000 rpm, 400 μL of the supernatant was taken out and added into 400 μL 10% TCA containing 0.67% (w/v) thiobarbituric acid (TBA) in a new tube, then the mixture was incubated in boiling water for 30 min. The mixture was centrifugated for 5 min at 12,000 rpm after cooling to room temperature and the OD_450 nm_, OD_532 nm_, and OD_600nm_ of the supernatant were determined using a Multimode Plate Reader (PerkinElmer).

The content of free proline in plants was determined according to a reported method [52]. Briefly, 100 mg fresh samples were homogenized in 1 mL 3% (w/v) sulfosalicylic acid using a Tissuelyser (Jingxin, Shanghai, China), and incubated in boiling water for 10 min. After cooling to room temperature, the samples were centrifugated at 12,000 rpm for 5 min. 400 μL of the supernatant was taken out and mixed with 400 μL acid ninhydrin and 400 μL glacial acetic in a new tube, then mixture was incubated in a boiling water bath for 30 min. After centrifuged at 6000 rpm for 5 min, the proline content was measured at 520 nm using a Multimode Plate Reader (PerkinElmer).

### The determination of ABA content

The quantitative analysis of the endogenous concentration of ABA was performed using an internal standard method as described previously [53]. ^2^H_6_ABA (OlChemIm, Olomouc, Czech Republic) was used as an internal standard.

### Dual-luciferase reporter assays in *Arabidopsis* protoplasts

The promoter regions of *AtNCED2*, *AtNCED5*, AtNCED6 and *AtNCED9* were amplified with the corresponding primers and inserted into pGreenII 0800 vector at *Hind*III and *BamH*I to obtain the *ProAtNCED2-*pGreenII 0800, *ProAtNCED5-* pGreenII 0800, *ProAtNCED6-* pGreenII 0800 and *ProAtNCED9-*pGreenII 0800 vectors as reporter constructs. The full length of *GhWRKY1-like* was amplified with *GhWRKY1-like*-SK-F/R and inserted into pGreenII 62-SK vector at *Pst*I and *BamH*I to obtain *GhWRKY1-like*-62-SK construct as an effector; the pGreenII 62-SK vector without any DNA insertion was used as an empty vector control. The dual-luciferase reporter assays in *Arabidopsis thaliana* (Col-0) protoplasts were performed as described previously [54].

## Supplementary Information

**Additional file 1: Figure S1.** Sequence and phylogenetic analysis of GhWRKY1-like. Sequence alignment of the amino acid sequence of GhWRKY1-like (XP_016696352) with AtWRKY1 (AEC05881), GmWRKY1 (XP_003518571), PtWRKY1 (XP_006375555), and TcWRKY1 (XP_007049283). Conserved WRKY domain and zinc finger motif are shown in red box and green box, respectively.

**Additional file 2: Figure S2.** The abiotic stresses including mannitol, ABA, dehydration and NaCl induced expression patterns of GhWRKY1-like.

**Additional file 3: Table S1.** Primers used in vectors constructed and qRT-PCR. The underlined sequences were the infusion sequences.

## Data Availability

All data generated or analyzed during this study are included in this published article and its supplementary information files.
