# Supplementary material for: GhWRKY1-like, a WRKY transcription factor, mediates drought tolerance in Arabidopsis via modulating ABA biosynthesis
Source: BMC Plant Biol. 2021 Oct 8;21:458. doi: 10.1186/s12870-021-03238-5 (PMC8501554; doi:10.1186/s12870-021-03238-5)
Supplement: Supplementary file 1 — Additional file 1: Figure S1. Sequence and phylogenetic analysis of GhWRKY1-like. Sequence alignment of the amino acid sequence of GhWRKY1-like (XP_016696352) with AtWRKY1 (AEC05881), GmWRKY1 (XP_003518571), PtWRKY1 (XP_006375555), and TcWRKY1 (XP_007049283). Conserved WRKY domain and zinc finger motif are shown in red box and green box, respectively. [file 12870_2021_3238_MOESM1_ESM.docx]

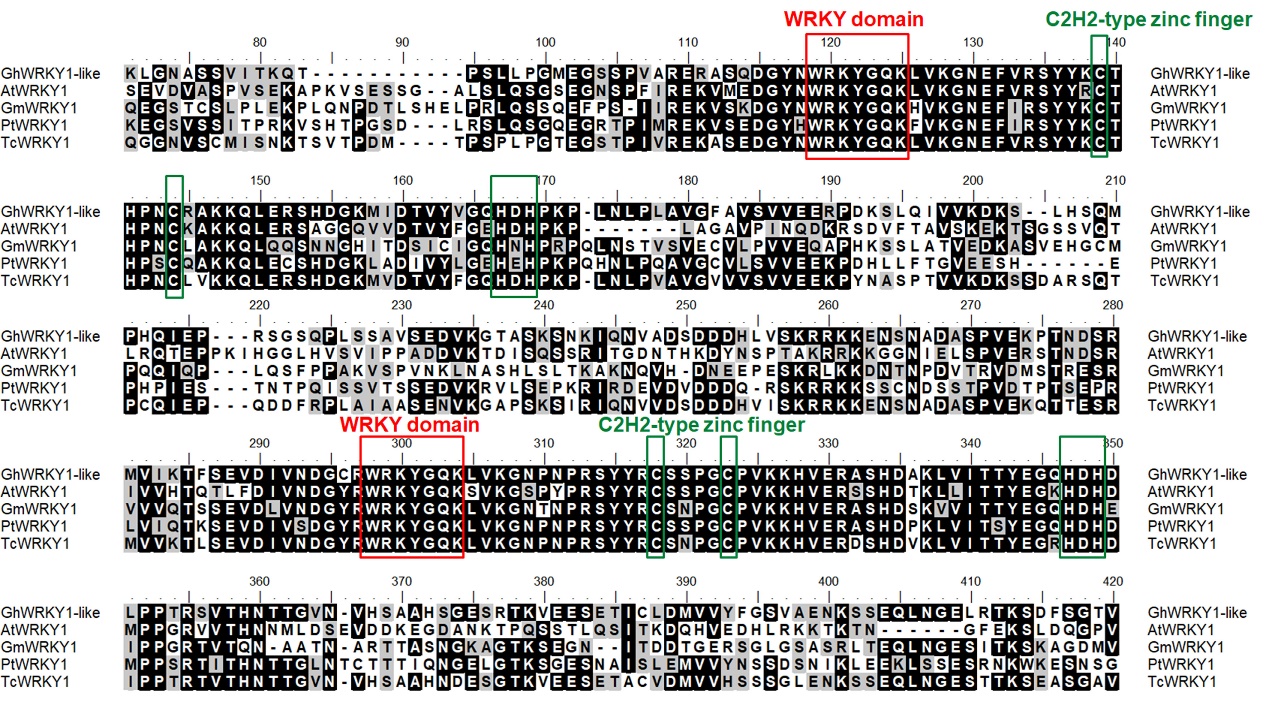


**Additional file 1: Figure S1. Sequence and phylogenetic analysis of GhWRKY1-like.** Sequence alignment of the amino acid sequence of GhWRKY1-like (XP_016696352) with AtWRKY1 (AEC05881), GmWRKY1 (XP_003518571), PtWRKY1 (XP_006375555), and TcWRKY1 (XP_007049283). Conserved WRKY domain and zinc finger motif are shown in red box and green box, respectively.
