# Supplementary material for: GhWRKY1-like, a WRKY transcription factor, mediates drought tolerance in Arabidopsis via modulating ABA biosynthesis
Source: BMC Plant Biol. 2021 Oct 8;21:458. doi: 10.1186/s12870-021-03238-5 (PMC8501554; doi:10.1186/s12870-021-03238-5)
Supplement: Supplementary file 2 — Additional file 2: Figure S2. The abiotic stresses including mannitol, ABA, dehydration and NaCl induced expression patterns of GhWRKY1-like. [file 12870_2021_3238_MOESM2_ESM.docx]

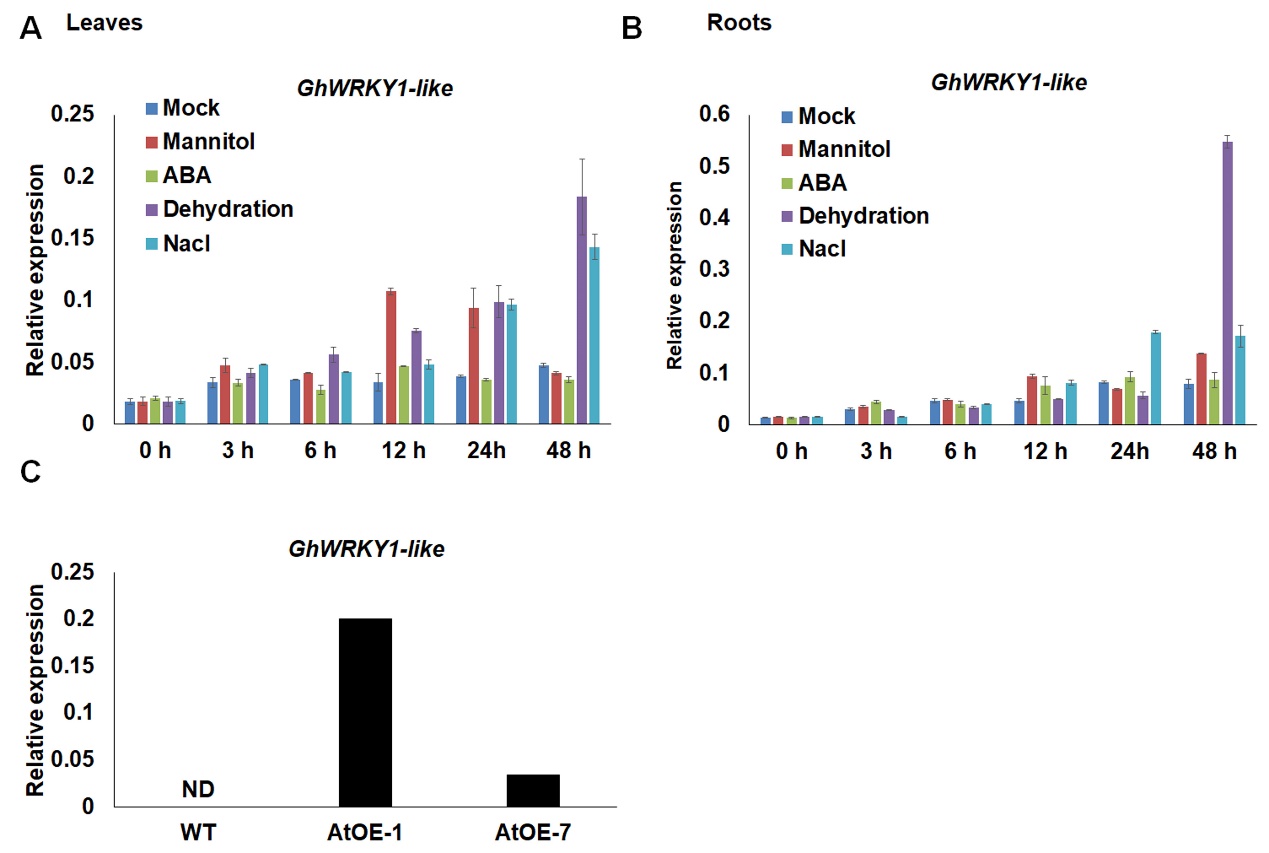


**Additional file 2: Figure S2. *GhWRKY1-like* is induced by different** **abiotic stresses in cotton. (A)** The induced expression pattern of *GhWRKY1-like* in the leaves of WT cotton line YZ1. **(B)** The induced expression pattern of *GhWRKY1-like* in the roots of WT cotton line YZ1. The values are normalized to *GhUB7* and expressed as the means ± SD; n = 3. **(C)** The expression levels of *GhWRKY1-like* of T3 transgenic lines analyzed through RT-qPCR. The values are normalized to *AtACTIN2* and expressed as the means ± SD; n = 3.
