# Supplementary material for: GhWRKY1-like, a WRKY transcription factor, mediates drought tolerance in Arabidopsis via modulating ABA biosynthesis
Source: BMC Plant Biol. 2021 Oct 8;21:458. doi: 10.1186/s12870-021-03238-5 (PMC8501554; doi:10.1186/s12870-021-03238-5)
Supplement: Supplementary file 3 — Additional file 3: Table S1. Primers used in vectors constructed and qRT-PCR. The underlined sequences were the infusion sequences. [file 12870_2021_3238_MOESM3_ESM.docx]

**Additional file 3: Table S1**

**Table S1.** Primary primers used in this study.

| Primer name Sequences (5'-3') Underlined | | | Destination |
| --- | --- | --- | --- |
| *qGhWRKY1-like*-F  *qGhWRKY1-like*-R  *GhWRKY1-like*-GFP-F  *GhWRKY1-like*-GFP-R  W-box-pHisi-1-F | AGACTCCCTCTCTGTTGCCTGG  TGTCAATCATTTTCCCATCGTG  GGGGACAAGTTTGTACAAAAAAGCAGGCTGGATGGTTCCGTCAGGGGAGTG  GGGGACCACTTTGTACAAGA AAGCTGGGTTAGGACCTGATATAGGTGCA  GAATTCCCGGGGAGCTCCGTTGACCGTTGACCGAGTTGACTTTTTTAGAGCTCACGCGTTCGCG | *att*B  *attP*  infusion | qRT-PCR  Subcellular Localization  Yeast one hybrid |
| W-box-pHisi-1-R | CGCGAACGCGTGAGCTCTAAAAAAGTCAACTCGGTCAACGGTCAACGGAGCTCCCCGGGAATTC | sequences |  |
| mW-box-pHisi-1-F  mW-box-pHisi-1-R | GAATTCCCGGGGAGCTCCGTAGACGGTAGACGGAGTAGACGTTTTTAGAGCTCACGCGTTCGCG  CGCGAACGCGTGAGCTCTAAAAACGTCTACTCCGTCTACCGTCTACGGAGCTCCCCGGGAATTC | infusion  sequences | Yeast one hybrid |
| *GhWRKY1-like*-AD-F  *GhWRKY1-like*-AD-R  *ProAtNCED2*-F  *ProAtNCED2*-R  *ProAtNCED3*-F  *ProAtNCED3*-R  *ProAtNCED5*-F  *ProAtNCED5*-R  *ProAtNCED6*-F  *ProAtNCED6*-R *ProAtNCED9*-F  *ProAtNCED9*-R  *ProAtNCED2*-pHisi-1-F  *ProAtNCED2*-pHisi-1-R  *ProAtNCED3*-pHisi-1-F  *ProAtNCED3*-pHisi-1-R  *ProAtNCED5*-pHisi-1-F  *ProAtNCED5*-pHisi-1-R  *ProAtNCED6*-pHisi-1-F  *ProAtNCED6*-pHisi-1-R  *ProAtNCED9*-pHisi-1-F  *ProAtNCED9*-pHisi-1-R  *GhWRKY1-like*-SK-F  *GhWRKY1-like*-SK-F  *ProAtNCED2*-0800-F  *ProAtNCED2*-0800-R  *ProAtNCED5*-0800-F  *ProAtNCED5*-0800-R  *ProAtNCED6*-0800-F  *ProAtNCED6*-0800-R  *ProAtNCED9*-0800-F  *ProAtNCED9*-0800-R  *qAtZEP* -F  *qAtZEP* -R  *qAtNCED2*-F  *qAtNCED2*-R  *qAtNCED3*-F  *qAtNCED3*-R  *qAtNCED5*-F  *qAtNCED5*-R  *qAtNCED6*-F  *qAtNCED6*-R  *qAtNCED9*-F  *qAtNCED9*-R  *qAtAAO3*-F  *qAtAAO3*-R  *qAtABA2*-F  *qAtABA2*-R  *qAtABI1*-F  *qAtABI1*-R  *qAtABI2-*F  *qAtABI2*-R  *qAtHAB1*-F  *qAtHAB1*-R  *qAtHAB2*-F  *qAtHAB2*-R  *qAtAHG1*-F  *qAtAHG1*-R  *qAtSnRK2.2*-F  *qAtSnRK2.2*-R  *qAtSnRK2.3*-F  *qAtSnRK2.3*-R  *qAtSnRK2.6*-F  *qAtSnRK2.6*-R  *qAtCPK3*-F  *qAtCPK3*-R  *qAtCPK4*-F  *qAtCPK4*-R  *qAtCPK6*-F  *qAtCPK6*-R  *qAtCPK11*-F  *qAtCPK11*-R  *qAtABF1*-F  *qAtABF1*-R  *qAtAREB3*-F  *qAtAREB3*-R  *qAtRD22*-F  *qAtRD22*-R  *qAtRD29A*-F  *qAtRD29A*-R  *qAtRD29B*-F  *qAtRD29B*-R  *qAtABI5*-F  *qAtABI5*-R  *qAtCOR15A*-F  *qAtCOR15A*-R  *qAtCOR15B*-F  *qAtCOR15B*-R  *qAtKIN1*-F  *qAtKIN1*-R  *qAtCER3*-F:  *qAtCER3*-R  *qAtLEA14*-F  *qAtLEA14*-R  *qAtLEA76*-F  *qAtLEA76*-R  *AtACTIN2*-F  *AtACTIN2*-R  *GhUB7*-F  *GhUB7*-R | AAAAGAGATCGAATTCATGGTTCCGTCAGGGGAGTGTG  GCAGGTCGACGGATCCTCAAGGACCTGATATAGGTGCA  ATTGTGCGAGTGTGAATGGGTTGG  GGCTTTTGTTTTCTTATCTTCGGT  GACAATATCGACCCTAATGTCCTT  TTTTCAAGTGTGTTCAATCAGTAT  AAATAAAGAGACCCAAAGACCAAA  GAGAGCAAATGACTTAAGAAAAGAAGC  TCCTTTGAAGCATCCTTGTCGTTT  GGTGGTGACTTGTGAGCGACCTAT  CTGAAATGGACACCTGAAGCCTGG  TCACGCTACTATTTTCTCATCTTT  GAATTCCCGGGGAGCTCATTGTGCGAGTGTGAATGGGTTGG  CGCGAACGCGTGAGCTCGGCTTTTGTTTTCTTATCTTCGGT  GAATTCCCGGGGAGCTCGACAATATCGACCCTAATGTCCTT  CGCGAACGCGTGAGCTCTTTTCAAGTGTGTTCAATCAGTAT  GAATTCCCGGGGAGCTCAAATAAAGAGACCCAAAGACCAAA  CGCGAACGCGTGAGCTCGAGAGCAAATGACTTAAGAAAAGA  GAATTCCCGGGGAGCTCTCCTTTGAAGCATCCTTGTCGTTT  CGCGAACGCGTGAGCTCGGTGGTGACTTGTGAGCGACCTAT  GAATTCCCGGGGAGCTCCTGAAATGGACACCTGAAGCCTGG  CGCGAACGCGTGAGCTCTCACGCTACTATTTTCTCATCTTT  CGCTCTAGAACTAGTGGATCCATGGTTCCGTCAGGGGAGTGTG  CTTGATATCGAATTCCTGCAGTCAAGGACCTGATATAGGTGCA  ACGGTATCGATAAGCTTATTGTGCGAGTGTGAATGGGTTGG  CTAGAACTAGTGGATCCGGCTTTTGTTTTCTTATCTTCGGT  ACGGTATCGATAAGCTTAAATAAAGAGACCCAAAGACCAAA  CTAGAACTAGTGGATCCGAGAGCAAATGACTTAAGAAAAGAAGC  ACGGTATCGATAAGCTTTCCTTTGAAGCATCCTTGTCGTTT  CTAGAACTAGTGGATCCGGTGGTGACTTGTGAGCGACCTAT  ACGGTATCGATAAGCTTCTGAAATGGACACCTGAAGCCTGG  CTAGAACTAGTGGATCCTCACGCTACTATTTTCTCATCTTT  GATTGCAGATTTTATACCAGCGGA  TACCATTTGGAGCATCAGCCCC  CTCCACTTCCCAAAACCGCTGA  TTGGCTCAAACATCGGATTCGC  GGCGGAGGGTTTCCTTGTCAGC  ATTTGACGGCGTGAACCATACC  CACGGTTGATGCTATTTTACGC  GGTCTCTAAATCGCCATTGTCA  GTATGAGCCCACCCGACACGAT  TATCTACCTTCGCAATGCCACT  TCACGGACATTTAGGAATCGCC  TTTTCGGGTGGGCTATCATTGT  CATTCAACAAGCGTATGGTCAG  TCCTTCGGTCTGTCCTAAATCA  GGGAGGCGTTGGTCCACATTCT  ATCAACCGTCAGTTCCACCCCT  GTTTGGGATGTAATGACGGATG  ACCACACTTATGTTGTCTTTGC  CGAGATCGATGAATCAGAGTGA  CCATCAAGCAACGAACTAGAAG  AGTAGTTTCTAACTGCGGTGAT  TTCAGATATCTGTCACCGATGG  TTGCACTTACTTTGGGTTTAGC  CAGCTGCATCAGTAACATTCTC  AGTTCGTCTAGGAATCGTGATC  TCCCATAACCGAAACTATACCG  TTAAAGAGGTGATTTTGACGCC  AATCCGCTCATAAAGTTCTCCA  ATTTCAAGAATCTTCGTGGCTG  CTGTGTTGCTCTCGTTCATTAG  ATATCTCCTGAATGTCGCCATT  GTTCATTAGATCTGCCGGTAGA  GGAAGTACGGAGAACCTATGAG  GTCTCTTTGTGAGTTACGAGGT  CGTAAGCTTGTATGTCGTGAAG  CAGCTTCACGTTCACTAAAACA  GACAAAATCTACGAGGGCAATC  TGTCCTTGTCCTAACTTACGAC  CCGATTTTGGTTTGTCTGTCTT  AGCACTCCACACATCTATTTCA  CCGAATCAAAACAGCATATCGT  CTGCGCTAGTCTCAAATAAACC  CAAAATCACTTGGGGAGTTCTG  ACCTCATCAACAGTCTTTTTGC  GACTTTCGATTTTACCGACGAG  CGCTACCGGTTTTACCTTTATG  TTCTGTAAGGACGACGTTTACA  CGTACTCGTTACATCCTCTGTT  GAAACCAAAGATGAGTCGACAC  TTTTTCGTAAACCGGAGTCAAC  AATAAGAGAGGGATAGCGAACG  GCTACCACCACCTCTATGTATC  CATTAGCAGATGGTGAGAAAGC  TCTCAGCTTCTTTACCCAATGT  GATGGCGAGAAAACAAAAGACT  CTTCTGCTTTACCCTCTACGAA  AACAAGAATGCCTTCCAAGC  CGCATCCGATACACTCTTTCC  GACTCATCGCATTAATTGTGCT  GTTACGATGGAAAGATCTGTGC  AGATTGGAAAGGGGAAGATACC  GATTTGGAGCTCGTAGTCAATG  GAGAAGACTGGACAAGCTATGG  TGGGCAGTTTGAGATGTCTTAT  GCTTCGTATTGCTCCTGAAG  GAAGAGAGAAACCCTCGTAG  GAAGGCATTCCACCTGACCAAC  CTTGACCTTCTTCTTCTTGTGCTTG | infusion  sequences  infusion  sequences  infusion  sequences  infusion  sequences  infusion  sequences  infusion  sequences  infusion  sequences  infusion  sequences  infusion  sequences  infusion  sequences  infusion  sequences | Yeast one hybrid  Promoter  cloning  Promoter  cloning  Promoter  cloning  Promoter  cloning  Promoter  cloning  Yeast one hybrid  Yeast one hybrid  Yeast one hybrid  Yeast one hybrid  Yeast one hybrid  Dual-Luciferase Report system  Dual-Luciferase Report system  Dual-Luciferase Report system  Dual-Luciferase Report system  Dual-Luciferase Report system  qRT-PCR    qRT-PCR  qRT-PCR  qRT-PCR  qRT-PCR  qRT-PCR  qRT-PCR  qRT-PCR  qRT-PCR  qRT-PCR  qRT-PCR  qRT-PCR  qRT-PCR  qRT-PCR  qRT-PCR  qRT-PCR  qRT-PCR  qRT-PCR  qRT-PCR  qRT-PCR  qRT-PCR  qRT-PCR  qRT-PCR  qRT-PCR  qRT-PCR  qRT-PCR  qRT-PCR  qRT-PCR  qRT-PCR  qRT-PCR  qRT-PCR  qRT-PCR  qRT-PCR  qRT-PCR  DQ116441 |
